# Photoacoustic imaging as a tool to probe the tumour microenvironment

**DOI:** 10.1242/dmm.039636

**Published:** 2019-07-16

**Authors:** Emma Brown, Joanna Brunker, Sarah E. Bohndiek

**Affiliations:** 1 Department of Physics, University of Cambridge, JJ Thomson Avenue, Cambridge CB3 0HE, UK; 2 Cancer Research UK Cambridge Institute, University of Cambridge, Li Ka Shing Centre, Robinson Way, Cambridge CB2 0RE, UK

**Keywords:** Optoacoustic imaging, Hypoxia, Cancer, TME, PAI, Vasculature

## Abstract

The tumour microenvironment (TME) is a complex cellular ecosystem subjected to chemical and physical signals that play a role in shaping tumour heterogeneity, invasion and metastasis. Studying the roles of the TME in cancer progression would strongly benefit from non-invasive visualisation of the tumour as a whole organ *in vivo*, both preclinically in mouse models of the disease, as well as in patient tumours. Although imaging techniques exist that can probe different facets of the TME, they face several limitations, including limited spatial resolution, extended scan times and poor specificity from confounding signals. Photoacoustic imaging (PAI) is an emerging modality, currently in clinical trials, that has the potential to overcome these limitations. Here, we review the biological properties of the TME and potential of existing imaging methods that have been developed to analyse these properties non-invasively. We then introduce PAI and explore the preclinical and clinical evidence that support its use in probing multiple features of the TME simultaneously, including blood vessel architecture, blood oxygenation, acidity, extracellular matrix deposition, lipid concentration and immune cell infiltration. Finally, we highlight the future prospects and outstanding challenges in the application of PAI as a tool in cancer research and as part of a clinical oncologist's arsenal.

## Abstract (precis)

**Summary:** This Review details the potential of photoacoustic imaging to visualise features of the tumour microenvironment such as blood vessels, hypoxia, fibrosis and immune infiltrate to provide unprecedented insight into tumour biology.

## Introduction

**Fig. 1.**
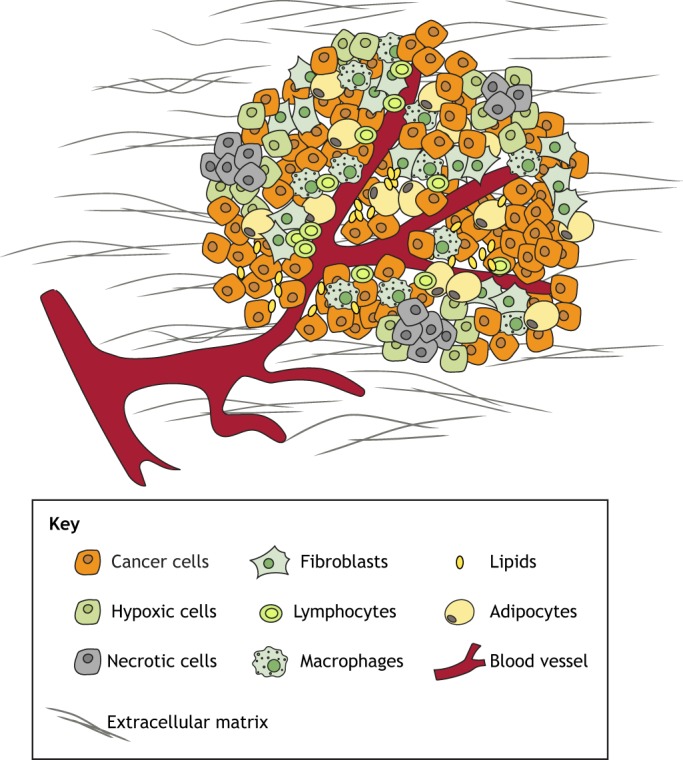
**The tumour microenvironment (TME).** Schematic diagram illustrating the involvement of multiple cell types in a tumour, including endothelial cells and pericytes that make up blood vessels, as well as immune cells, fibroblasts and adipocytes, alongside the cancer cells. Lipids are synthesised by adipocytes and cancer cells. Hypoxia arises as the tumour grows beyond the limit of oxygen diffusion from the surrounding vessels. Fibrosis arises from excessive deposition of extracellular matrix (ECM) components without concurrent degradation. A supportive environmental niche of these chemical and physical signals evolves with the cancer cells to promote tumour development and progression.

The focus of cancer research has moved from treating the tumour as a homogenous mass of cancer cells to considering the wider tumour microenvironment (TME; see Box 1 for a glossary of terms) (Quail and Joyce, 2013). The infrastructure of a growing solid tumour is a heterogeneous mixture of cancer and stromal cells. In addition to this complex cellular picture, chemical signals such as hypoxia (Box 1) and physical signals arising from fibrosis (Box 1), among others (Fig. 1), are linked to poor patient prognosis (Gilkes et al., 2014). Importantly, these features of the TME interact with and regulate one another (De Palma et al., 2017; Kalluri, 2016; LaGory and Giaccia, 2016), and such dynamic relationships influence tumour growth and heterogeneity.

**Box 1. Glossary**

**Absorption coefficient:** the fraction of incident radiant energy absorbed per unit mass or thickness of an absorber.

**Angiogenesis:** the development of new blood vessels from pre-existing vessels. Driven by pro-angiogenic factors such as VEGF and inhibited by anti-angiogenic factors.

**Contrast agent:** a substance or molecule that is administered into a living subject to enhance the visualisation of a particular structure or biological process within the body in medical imaging.

**Extracellular matrix (ECM):** a three-dimensional, non-cellular structure produced and secreted by cells. It is composed primarily of fibrous elements such as collagen and forms the interstitial connective tissue and the basement membrane.

**Fibrosis:** the thickening and scarring of connective tissue, usually as a result of injury.

**Hypoxia:** a deficiency in the amount of oxygen reaching tissues.

**Isosbestic point:** a wavelength at which the absorption coefficient of two molecules is equal.

**Light fluence:** optical energy per unit area.

**Optical diffusion limit:** the depth in biological tissue beyond which light propagating along the predefined linear trajectory becomes too weak to be detected in practice (Wang and Hu, 2012).

**Perfusion:** the flow rate of blood per mass of tissue.

**Photoacoustic (optoacoustic) imaging (PAI):** a biomedical imaging modality based on the photoacoustic effect.

**Photoacoustic effect:** the generation of sound waves following light absorption in a material such as tissue.

**Tumour microenvironment (TME):** the cellular environment in which a tumour exists, including blood vessels, fibroblasts, immune cells and the ECM.

Improving our understanding of the role of the TME in cancer progression would strongly benefit from non-invasive visualisation of the tumour as a whole organ *in vivo*, both preclinically in mouse models of the disease, as well as in patients. Unfortunately, features of the TME remain challenging to resolve with non-invasive imaging. As a result, many studies still rely on excised tissues *ex vivo*, which only interrogate a small portion of the tumour at a fixed time point, and are not able to capture its full spatial and temporal heterogeneity. Visualising the dynamic TME *in vivo* would allow researchers to investigate key questions about the interplay between different features of the microenvironment, for example, the functional relationship between hypoxia and fibrosis (Gilkes et al., 2014). The biomarkers resulting from these studies could then be applied clinically to predict tumour aggressiveness, stratify patients and monitor treatment response. Ultimately, this would transform patient care and improve survival by helping to guide therapeutic strategies (Abadjian et al., 2017; Weissleder et al., 2016).

A range of existing *in vivo* imaging techniques can be used to visualise different facets of the TME and have already provided valuable insight. Unfortunately, they come with limitations that include limited spatial resolution, extended scan times and poor specificity from confounding signals. Photoacoustic (also referred to as optoacoustic) imaging (PAI; Box 1) is a promising technique with potential to overcome these limitations for imaging TME features *in vivo*. This Review will first introduce key aspects of the TME, focusing on tumour vasculature and hypoxia, the extracellular matrix (ECM; Box 1) and lipids, together with the immune cell compartment, explaining their individual importance in cancer biology and their dynamic interactions as a microenvironment. We will then review the advantages and disadvantages of current techniques for imaging these key components *in vivo* and specifically focus on the potential of PAI in such studies. We conclude by discussing the potential of PAI for clinical imaging of the TME and the outstanding challenges that must be overcome to achieve this.

## Features of the TME

### Tumour vasculature and hypoxia in the TME

A growing tumour mass requires a vascular network to supply cancer cells with nutrients and remove metabolic waste products, which then permits cancer cells to extravasate and metastasise. Development of a vascular network typically begins in response to diffusion-limited or chronic hypoxia, which arises when the diffusion of oxygen from surrounding blood vessels is insufficient to meet the demand of the proliferating cancer cells (LaGory and Giaccia, 2016; Lundgren et al., 2007). Activation of hypoxia-inducible factors (HIFs) drives the transcription of genes involved in a wide range of cellular functions (LaGory and Giaccia, 2016; Lundgren et al., 2007), including the production of pro-angiogenic factors, such as vascular endothelial growth factor (VEGF), that stimulate endothelial cells to proliferate, sprout and form new blood vessels (Folkman, 1971; Hoeben et al., 2004). Often an imbalance of pro- and anti-angiogenic factors then occurs, which results in a chaotic and heterogeneous network of blood vessels, including many immature vessels with poor pericyte coverage (Ribeiro and Okamoto, 2015), irregular branching and a tortuous morphology (Hanahan and Weinberg, 2011; Krishna Priya et al., 2016; Nagy and Dvorak, 2012; Nagy et al., 2007).

Several important prognostic consequences arise from these features of the tumour vasculature. Firstly, perfusion-limited (Box 1), or cycling, hypoxia occurs in cancer and stromal cells adjacent to the immature tumour blood vessels, generating repeated dynamic cycles of ischaemia and reperfusion that produce oxidative stress, which leads to increased stem-cell-like properties of cancer cells and resistance to therapy (Michiels et al., 2016). Secondly, heterogeneity of vessel perfusion leads to unequal access of systemic therapeutic agents within the tumour. Although some areas of the tumour – those that are well perfused – will likely receive the expected therapeutic dose, other poorly perfused areas may receive a lower dose, meaning that they can survive the treatment (Trédan et al., 2007). Finally, as tumours shift to a predominantly glycolytic metabolism via the expression of HIF target genes, poor perfusion allows lactic acid (from anaerobic glycolysis) and protons (from the conversion of CO_2_ produced from the pentose phosphate pathway) to accumulate in the TME, giving rise to acidosis (Parks and Pouysségur, 2017; Parks et al., 2010). Acidosis can increase cancer cell invasion (Estrella et al., 2013) and metastatic potential (Rofstad et al., 2006), and decrease drug efficacy (Vukovic and Tannock, 1997). The HIF1α, VEGF and associated pathways are linked to poor prognosis in a range of solid tumours (Hegde et al., 2013; Lundgren et al., 2007; Semenza, 2016), demonstrating the consequences of tumour hypoxia on patient outcome.

### ECM and lipid composition of the TME

The tissue ECM is a three-dimensional, non-cellular structure composed primarily of collagen, which forms the interstitial connective tissue and the basement membrane. ECM function extends beyond physical support to directly influence cell growth and motility. Dysregulation of ECM remodelling in cancer leads to excessive production and deposition of ECM proteins, termed fibrosis (Lu et al., 2012), which drives tumour progression by: stimulating cancer cell proliferation (Levental et al., 2009); changing cancer cell contractility, polarity and mechanosignalling (Gilkes et al., 2014); providing a reservoir of growth factors and pro-angiogenic factors (Bonnans et al., 2014; De Palma et al., 2017); and promoting epithelial-to-mesenchymal transition (EMT) (Zhang et al., 2013). It is therefore unsurprising that increased expression of ECM remodelling genes, particularly in breast cancer, leads to higher mortality (Chang et al., 2005). Although the absence of fibrotic foci does not necessarily indicate benignity, highly fibrotic breast tumours have a poor prognosis (Conklin et al., 2011), with a high risk of recurrence (Hasebe et al., 1997).

The lipid content of tumours is significantly different from that of healthy tissues and plays an important role in the TME, particularly in lipid-rich tissues such as the breast (Baenke et al., 2013; Choi et al., 2018). Fatty acids, arising from adipocytes or synthesised by cancer cells themselves in *de novo* lipogenesis, can provide cancer cells with an alternative energy source in times of chronic nutrient depletion, which may be spatially heterogeneous and relates to the state of tumour perfusion (Baenke et al., 2013; Choi et al., 2018; Kuhajda et al., 1994; Michiels et al., 2016; Walter et al., 2009). In addition to providing an alternative energy source, lipids have structural roles in phospholipid membranes, which are needed for cell proliferation, and lipid mediators such as prostaglandin E2 promote cancer cell proliferation and survival via MAPK signalling (Krysan et al., 2005).

### Cellular components of the TME

The presence of endothelial cells, adipocytes and fibroblasts in the TME serve to define the aforementioned environmental features. Cancer-associated fibroblasts (CAFs) are the predominant stromal cell type secreting ECM in the TME and have a complex role that has been extensively reviewed elsewhere (Kalluri, 2016; LeBleu and Kalluri, 2018). It is worth noting that, in addition to ECM production, CAFs secrete matrix metalloproteases (MMPs) (reviewed in LeBleu and Kalluri, 2018). MMPs break down ECM and basement membrane, promoting cancer cell invasion and EMT. Endothelial cells also secrete MMPs to allow sprouting and angiogenesis (Box 1) (reviewed in Egeblad and Werb, 2002; Kalluri, 2016).

However, a major cellular component that merits discussion is the immune cell infiltrate. It is now well known that immune cells play a vital role in tumour development (Fridman et al., 2017; Joyce and Fearon, 2015), which has led to the recent emergence of immunotherapy strategies (reviewed in Rosenberg and Restifo, 2015; Sharma and Allison, 2015). The dual role of immune cells in the TME is exemplified by the fact that cancer cells evade the immune system in order to survive (Sharma and Allison, 2015), while, on the other hand, many immune cell types release immunosuppressive cytokines and act in a pro-tumorigenic manner (Box 2).

**Box 2. The immune system in the tumour microenvironment**

**Anti-tumour immunity**

Immune cells can recognise cancer cells as foreign and initiate an immune response against them (see the following reviews for more detail: Blomberg et al., 2018; Joyce and Fearon, 2015). This is normally initiated in the same way the immune system recognises and kills foreign pathogens. A tumour-specific antigen released by cancer cells is taken up by dendritic cells (DCs), which are part of the innate immune system. Under pro-inflammatory signals from the tumour microenvironment (TME), DCs migrate to the nearest lymph node, where they interact with and activate T cells, which are part of the adaptive immune system. CD8+ cytotoxic T cells specific to the tumour antigen migrate from the lymph node back to the tumour to kill cancer cells that express the antigen. Ideally, the adaptive immune response should generate immunological memory, meaning that the CD8+ T cells will expand and kill any future cancer cells expressing the same tumour-specific antigen. This is in contrast to innate immune cells, which generally recognise foreign antigens and activate the adaptive immune system. Innate immune cells such as natural killer cells can also kill cancer cells. Unfortunately, cancer cells develop many mechanisms to evade anti-tumour immunity and survive, for example by expressing immune checkpoint receptors. When these receptors bind to CD8+ T cells, they cause the T cell to become deactivated (anergic) and unable to carry out its cytotoxic functions. Immunotherapies such as checkpoint receptor inhibitors aim to increase anti-tumour immunity by blocking these immunosuppressive signals (Sharma and Allison, 2015).

**Pro-tumour immunity**

Cancer cells can also evade the anti-tumour immune response via pro-tumour immune cells. Cytokines such as TGF-β and IL-10 released by cancer cells or other stromal cells such as cancer-associated fibroblasts (CAFs) can skew CD4+ T-helper cells to become immunosuppressive and begin to inhibit the effect of cytotoxic cell types. Innate immunosuppressive cells such as tumour-associated macrophages (TAMs) are also anti-inflammatory and pro-angiogenic, driving tumour progression (Blomberg et al., 2018). TAMs are often considered to have a classical ‘M2’ anti-inflammatory phenotype, although there are some differences in gene expression (Franklin et al., 2014). This is in contrast to the pro-inflammatory ‘M1’ phenotype; however, the subsets of macrophages in the TME should be considered as more of a spectrum of phenotypes, rather than two distinct groups (Hobson-Gutierrez and Carmona-Fontaine, 2018).

Monitoring tumour response to immunotherapy is currently a major clinical challenge due to the dynamic nature of the immune TME. To optimise immunotherapy use whilst minimising toxicity and cost in non-responders, appropriately validated non-invasive imaging techniques could be applied to monitor the evolution of the TME in response to immunotherapy in real time and avoid repeated tissue sampling (Gibson et al., 2018; Mehnert et al., 2017; Schuurhuis et al., 2009; Tavaré et al., 2014). There is currently no single accurate biomarker to stratify patients for immunotherapy; it is likely that multiple biomarkers will need to be used together to give an accurate representation of a patient's TME (Gibney et al., 2016; Mehnert et al., 2017).

### Dynamic interactions of TME features

It is increasingly evident that features of the TME regulate each other. Hypoxia plays a central role in these interactions. For example, increased deposition of ECM proteins and recruitment of stromal cells leads to fibrotic contraction of the interstitial tissue space, which is associated with: increased interstitial fluid pressure (Heldin et al., 2004); the collapse of immature blood vessels; and restricted oxygen diffusion, which causes hypoxia (Jain et al., 2014). Conversely, at the molecular level, HIF1α directly activates transcription of genes required for collagen synthesis and cross-linking, such as procollagen lysyl hydroxylase 2 (Erler et al., 2006; Gilkes et al., 2013a,b), which increases angiogenic signalling and vessel permeability (Bordeleau et al., 2017; Saupe et al., 2013). This could actually result in decreased oxygen delivery to the tumour and thus further hypoxia.

The pro-angiogenic signalling of many stromal cell types is also hypoxia-mediated. Hypoxia drives macrophage polarisation towards an M2 phenotype (Box 2), which is anti-inflammatory and pro-angiogenic (Colegio et al., 2014). Many M2 macrophages are Tie2^+^ and cluster around existing blood vessels, promoting angiogenesis (Britto et al., 2018; De Palma et al., 2003, 2005; Hughes et al., 2015) and vessel permeability (Stockmann et al., 2008). The milieu of cytokines and growth factors secreted by CAFs contain further pro-angiogenic factors (De Palma et al., 2017), while hypoxia upregulates the production of sphingosine lipids (Ahmad et al., 2006; Pyne and Pyne, 2010) that are also likely to drive angiogenesis (Baenke et al., 2013; Nakajima et al., 2017). Additionally, hypoxia and acidosis modulate the function of all immune cell types, meaning that hypoxic niches spatially fine-tune the TME (Calcinotto et al., 2012; LaGory and Giaccia, 2016).

Finally, many therapies modulate the evolution of the TME and the dynamic relationships within. Anti-angiogenic treatments, which often target the VEGF pathway, have been shown to ‘normalise’ tumour vasculature by increasing vessel maturity, leading to re-oxygenation of the tumour and decreased hypoxia in mouse models (Dickson et al., 2007; Winkler et al., 2004). As mentioned above, immunotherapies such as checkpoint receptor inhibitors (Box 2) aim to overcome the negative regulation of immune cells in the TME (Joyce and Fearon, 2015) and have been particularly successful in melanoma patients displaying a large immune infiltrate (Hodi et al., 2010).

These dynamic relationships paint a complicated picture of the TME that will be explored for many years to come. Being able to monitor the dynamics of the TME *in vivo* is therefore essential to improve our understanding of tumour biology and ultimately improve treatments for patients.

## Visualising the TME

### Existing imaging modalities

Several preclinical and clinical imaging techniques are already available to probe various features of the TME (Table 1). Vascular perfusion can be visualised using intravenous administration of a contrast agent through ‘dynamic contrast-enhanced’ approaches (Box 1) (Alonzi et al., 2007; Ohno et al., 2014; Saini and Hoyt, 2014), which, despite being widely used, do have some associated toxicity concerns (Heiken, 2008; Rogosnitzky and Branch, 2016).

**Table 1.**
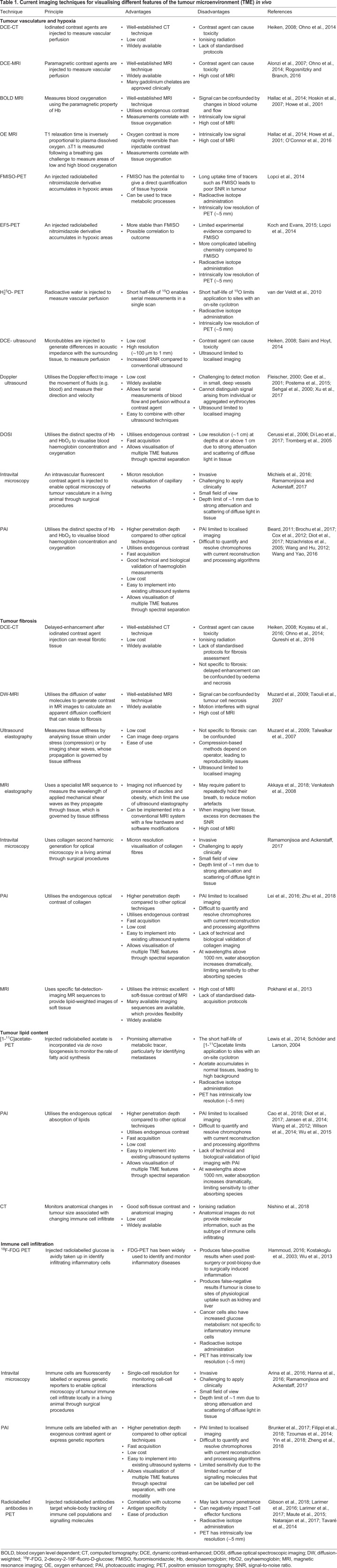
**Current imaging techniques for visualising different features of the tumour microenvironment (TME) *in vivo***

Hypoxia represents a challenge for imaging in medical diagnostics, as the method must generate a positive signal for the absence of oxygen. Preclinical studies using intravital imaging have revealed blood flow fluctuations and perivascular changes in hypoxia (Michiels et al., 2016), but these approaches have limited prospect for routine clinical use because they are invasive (Table 1). Magnetic resonance imaging (MRI)-based approaches such as blood-oxygen-level-dependent (BOLD) MRI (Hoskin et al., 2007) and oxygen-enhanced (OE) MRI (O'Connor et al., 2016) correlate with tissue oxygenation and histological markers of hypoxia (Hoskin et al., 2007; O'Connor et al., 2016). However, they suffer from intrinsically low sensitivity (Hallac et al., 2014; Howe et al., 2001). Positron emission tomography (PET) agents for hypoxia visualisation, including those derived from nitroimidazole, are also available (Koch and Evans, 2015; Lopci et al., 2014), but application to studying the TME is limited by the inherently low spatial resolution of PET and the requirement to administer a radiopharmaceutical. Diffuse optical spectroscopic imaging (DOSI) is a low-cost and readily accessible approach that measures local optical absorption coefficient (Box 1). DOSI can measure concentrations of oxy- and deoxy-haemoglobin (HbO_2_ and Hb, respectively) as surrogate markers of hypoxia (Cerussi et al., 2006; Di Leo et al., 2017; Tromberg et al., 2005) but has poor resolution at depths beyond ∼1 mm due to light scattering in tissue (see ‘optical diffusion limit’ in Box 1) (Wang and Hu, 2012).

A number of modalities can image fibrosis and lipids (Table 1). Dynamic contrast-enhanced (DCE)–computed-tomography (CT) is clinically approved for imaging tumour fibrosis but it measures interstitial tissue volume, so can be confounded by oedema and necrosis (Koyasu et al., 2016). Diffusion-weighted (DW) MRI, as well as ultrasound and MR-based elastography methods, can image fibrosis but are non-specific and generally have low sensitivity (Muzard et al., 2009; Talwalkar et al., 2007; Taouli et al., 2007). Ultrasound elastography can also have technical failures if obesity or ascites are present, although using MRI elastography instead may overcome this problem (Akkaya et al., 2018). Lipids can be detected in tumours by using MRI (Pokharel et al., 2013), and imaging the rate of fatty acid synthesis is possible with PET agents (Lewis et al., 2014; Schöder and Larson, 2004), but, due to the disadvantages described above, PET is not used regularly.

Preclinical tracking of CAFs, the predominant ECM-producing cell type (Kalluri, 2016), has been achieved using a PET tracer to target fibroblast activation protein (FAP) expressed on CAFs (Giesel et al., 2019), and intravital microscopy in transgenic mice with fibroblasts expressing a green fluorescent reporter protein (Arina et al., 2016). Tracking cells *in vivo* with reporter gene technology is ideal, as daughter cells will also express the reporter, meaning the contrast agent is not diluted. However, as it requires genetic manipulation, reporter gene technology has not yet been translated into clinical applications (Ramamonjisoa and Ackerstaff, 2017).

Despite overwhelming evidence for the importance of immune cells in tumour biology (Fridman et al., 2017; Joyce and Fearon, 2015), there is currently no clinical *in vivo* imaging method approved to visualise immune cells in the TME. Intravital microscopy and PET are most commonly employed in the research setting (Table 1). Intravital imaging of immune cells expressing a fluorescent reporter gene provides single-cell resolution and has revealed new immune-cell–tumour-cell interactions in preclinical models (Hanna et al., 2015, 2016). ^18^F-fluorodeoxyglucose PET is used to monitor tumour glucose metabolism and could also monitor inflammatory cell infiltration, as these cells are also glycolytic (Hammoud, 2016; Kostakoglu et al., 2003; Wu et al., 2013). Researchers also developed PET agents to specifically track immune cell populations and cytokines and monitor response to immunotherapy (Gibson et al., 2018; Larimer et al., 2016, 2017; Maute et al., 2015; Natarajan et al., 2017; Tavaré et al., 2014). Radiolabelled antibodies have been used to label T-cell populations (Larimer et al., 2016; Natarajan et al., 2017; Tavaré et al., 2014) as these cells infiltrate the tumour and can predict immunotherapy response (Larimer et al., 2016). In the clinic, anatomical CT images of patients on immunotherapy have shown a ‘pseudoprogression’ effect, whereby tumour size initially increases due to abundant T-cell infiltrate and, later, tumour size decreases in responders (Nishino et al., 2018).

Notably, many of the techniques described here suffer from limited spatial resolution, poor specificity from confounding signals and the need to administer contrast agents (Table 1). Additionally, none of the techniques described reveal multiple features of the TME within the same imaging scan. Mapping the dynamic interactions of TME features is paramount to our understanding of tumour biology and could have significant clinical applications. Hence, there remains an unmet clinical need for validated imaging biomarkers of the TME that can be measured cost-effectively at high spatial and temporal resolution. PAI could offer the flexibility to monitor multiple features with one modality using multi-wavelength imaging, providing a more complete picture of the TME.

### Photoacoustic imaging

**Fig. 2.**
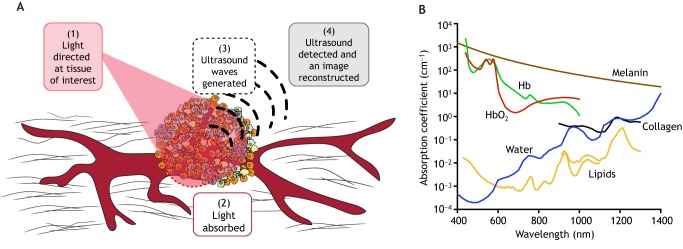
**Principles of photoacoustic imaging (PAI).** (A) During PAI, pulses of light illuminate the tissue (1). When light is absorbed (2), a transient heating gives rise to ultrasound waves (3). The ultrasound waves are then detected and used to reconstruct an image of the optical absorption in tissue (4). (B) Absorption spectra of endogenous molecules that absorb light pulses and can provide insight into the tumour microenvironment (TME). Panel B is reproduced with permission from Weber et al. (2016). This image is not published under the terms of the CC-BY licence of this article. For permission to reuse, please see Weber et al. (2016). Hb, deoxyhaemoglobin; HbO_2_, oxyhaemoglobin.

PAI is an emerging imaging modality, currently in clinical trials (Knieling et al., 2017; Steinberg et al., 2019), that could improve our visualisation of the TME. To create an image, the tissue of interest is illuminated with pulses of light, which cause a pressure change when absorbed and generate ultrasound waves that are detected at the tissue surface using one or more detectors (see ‘Photoacoustic effect’ in Box 1; Fig. 2A) (Beard, 2011; Ntziachristos et al., 2005; Wang and Yao, 2016). A major advantage of PAI is its scalability: by selecting different light sources, ultrasound detectors and scanning methods, it is possible to tune the spatial resolution, temporal resolution, imaging depth and image contrast. Both image spatial resolution and tissue attenuation scale with increasing ultrasound frequency: low-frequency ultrasound detection at a few MHz enables deep-tissue imaging with centimetre penetration and submillimetre resolution, whereas increasing the detection frequency pushes towards micron resolution but compromises the penetration depth. Technical developments in data acquisition and processing speeds will lead to improvements in the temporal resolution, but current state-of-the-art technology can achieve sub-second two-dimensional imaging, and three-dimensional images in seconds to minutes (Beard, 2011; Ntziachristos et al., 2005; Wang and Yao, 2016). To form an image, ultrasound signals must be acquired from different locations, either by scanning a single detector over the region of interest or by employing a detector array, and then these signals can be naively backprojected (beamformed) or subjected to more complex reconstruction algorithms to improve image quality (Beard, 2011).

Photoacoustic image contrast arises due to optical absorption, which results in ultrasound generation. When using near-infrared wavelengths of light (620-950 nm) for illumination, several vital endogenous molecules for the TME strongly absorb light (Fig. 2B). Using only endogenous contrast and acquiring data at multiple wavelengths, PAI can therefore non-invasively visualise vascular morphology, blood oxygenation, fibrosis and lipid content simultaneously with a single technique. Other features, such as immune cell infiltration, can be imaged by introducing targeted exogenous contrast agents or *in-vitro-*labelled immune cells, allowing a complete picture of the dynamic relationships in the TME to be revealed with a single technique. Such contrast agents, either injected externally, taken up by cells (Weber et al., 2016) or expressed in genetically modified cells (Brunker et al., 2017), work by providing additional sources of optical absorption with distinct spectra; the contrast agent is typically bound to, or expressed by, a targeting component in order to highlight specific structural or functional tissue features. PAI with exogenous contrast agents remains subject to the same spatial resolution and penetration depth trade-off as imaging of endogenous molecules; in addition, it is important to select a signalling compound with distinct absorption peaks and strong optical absorption above 600 nm, thus avoiding signal corruption by endogenous molecules (Fig. 2B).

Compared to existing optical techniques such as DOSI and intravital microscopy, PAI maintains high spatial resolution to a greater imaging depth due to the detection of ultrasound waves, which scatter less than light in tissue. By utilising optical absorption and contrast, PAI maintains high molecular specificity (Beard, 2011; Ntziachristos et al., 2005; Wang and Yao, 2016) compared to standard ultrasound techniques (Table 1). Additionally, many TME features can be detected simultaneously with PAI due to the multi-wavelength data acquisition, without administration of a contrast agent or radioactive agent. Despite the ability of PAI to image beyond the optical diffusion limit (Box 1), it has limited penetration depth compared to clinical whole-body modalities such as MRI, PET and CT. Preclinically, PAI is frequently used as a whole-body imaging modality, but this is not possible clinically, where the depth limits of PAI are approached. Introducing PAI through endoscopes can lift this limitation to some extent, but PAI would not compete with whole-body modalities such as PET for disease staging in patients (Table 1). Nonetheless, in tumours growing within the depth-detection limits of PAI, such as in the breast, PAI could follow initial conventional diagnostic imaging with MRI or ultrasound to provide additional insight into the TME. It is worth noting that combining imaging in a multi-modal approach would allow the limitations of one technique to be compensated for by another, and it is likely that PAI will be used in this way, for example by combining PAI with ultrasound (Bar-Zion et al., 2016; Diot et al., 2017; Neuschler et al., 2018). For the remainder of this Review, we will discuss how PAI has been used so far to image the TME, some of the challenges currently faced, and its potential for preclinical and clinical application in oncology.

## Photoacoustic imaging of vasculature and oxygenation

**Fig. 3.**
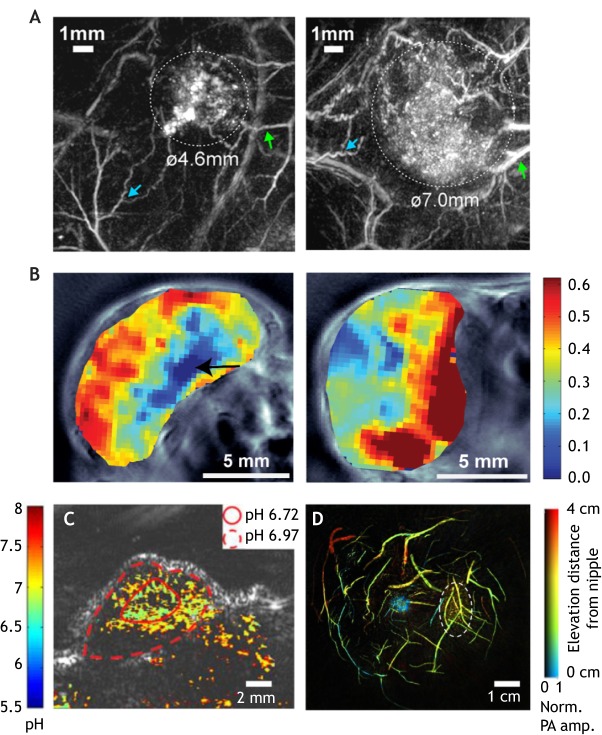
**Example photoacoustic images of the vasculature.** (A) Shown are *x*-*y* maximum intensity projections of a human colorectal tumour (SW1222) and the surrounding vasculature between day 7 and day 8 post-inoculation. Dashed white lines indicate tumour margins. Green arrows show common vascular features between images. Increasing tortuosity of normal blood vessels between day 7 and 8 is indicated by blue arrows. Reproduced with permission from Laufer et al. (2012) and the *Journal of Biomedical Optics*. This image is not published under the terms of the CC-BY licence of this article. For permission to reuse, please see Laufer et al. (2012). (B) Representative images of PC3 (left) and LNCaP (right) tumours showing the spatial distribution of ΔsO_2_ measured using PAI at multiple wavelengths. PC3 tumours displayed lower ΔsO_2_ compared to LNCaP tumours and had a core with low ΔsO_2_ (black arrow). Reproduced with minor formatting changes from Tomaszewski et al. (2017). (C) Photoacoustic pH image of rat glioma tumours at 75 min after SNARF-5F nanoparticle injection. The pH in the centre area (i.e. the area within the solid line) and the peripheral areas (i.e. the area between the solid line and the dashed line) are averaged, respectively. Reproduced with minor formatting changes from Jo et al. (2017). (D) Depth-encoded PAI images of the breast acquired while the patient, a 49-year-old woman with a stromal ﬁbrosis or ﬁbroadenoma, held her breath. Dashed white lines indicate tumour margin. Reproduced with minor formatting changes from Lin et al. (2018).

Preclinical PAI in small animal models allows detailed tumour vascular architecture and morphology to be visualised non-invasively over time at sub-100-μm resolution using just a single wavelength of light, commonly selected as an isosbestic point (Box 1) of Hb and HbO_2_ (e.g. 532 nm). This method has been used to monitor the developing vasculature in early tumours, showing changes in structure such as increased tortuosity (Fig. 3A) (Laufer et al., 2012), diameter and density (Lao et al., 2008), as well as the recruitment of existing vessels to feed the tumour mass (Laufer et al., 2012; Omar et al., 2015). Imaging biomarkers such as blood volume and vessel connectivity could be extracted from these images, but the majority of studies to date did not include quantification. Nonetheless, they show how PAI can provide high-resolution visualisation of the development of tumour vasculature at depths of several centimetres.

Alternatively, utilising the differential absorption spectra of Hb and HbO_2_ (Fig. 2B), PAI data can be recorded at multiple wavelengths and subjected to spectral unmixing algorithms to calculate imaging biomarkers related to total haemoglobin concentration (THb=Hb+HbO_2_) and blood oxygen saturation (sO_2_=HbO_2_/THb). These functional parameters can provide further insight into the TME, as detailed below.

PAI THb tends to be higher in tumours compared to normal tissue (Chekkoury et al., 2016; Li et al., 2008; Raes et al., 2016) because of increased angiogenesis, and also tends to be concentrated around the periphery as the cell line models used, such as melanoma cell-line-derived xenografts (Lavaud et al., 2017), tend to be less vascularised in their core. One study demonstrated a decrease in THb as the tumour developed (Imai et al., 2017), showing that this parameter may be context-dependent and that PAI can sensitively resolve such differences.

PAI generally measures lower sO_2_ values in tumours compared to normal tissue (Chekkoury et al., 2016; Imai et al., 2017; Lavaud et al., 2017; Li et al., 2008; Raes et al., 2016), consistent with poor perfusion and/or high consumption of O_2_ from the blood due to tumour hypoxia. A recent study in breast cancer models demonstrated that sO_2_ measurements correlated with vascular maturity, measured by pericyte coverage of vessels *ex vivo* (Quiros-Gonzalez et al., 2018). Altering the gas delivered to the mouse from air to 100% oxygen and measuring the change in sO_2_ can distinguish between well-perfused (with high ΔsO_2_) and poorly perfused (with low ΔsO_2_) prostate cancer models (Fig. 3B) (Bendinger et al., 2018; Tomaszewski et al., 2017). Importantly, low sO_2_ and ΔsO_2_ spatially correlate with regions of tissue hypoxia and necrosis (Gerling et al., 2014; Tomaszewski et al., 2018). In the same studies, vascular perfusion was measured *in vivo* using Indocyanine Green, a clinically approved near-infrared dye. In general, functional PAI studies have focussed on blood flow (van den Berg et al., 2015) rather than perfusion, and measuring blood flow at clinically relevant depths has proved to be challenging (Brunker and Beard, 2016) due to a poor velocity signal-to-noise ratio (SNR). However, it is possible that alternative approaches (for example, speckle tracking) could provide semi-quantitative flow/perfusion information that, combined with measures of blood oxygenation, could indicate the level of oxygen delivery to the tumour.

PAI biomarkers relating to vascular function have also been used to predict and monitor treatment response in preclinical cancer models. A decrease in THb and a corresponding increase in HbO_2_ has been seen in ovarian mouse tumour models following anti-angiogenic therapies, indicating vessel normalisation, which was shown *ex vivo* by an increase in pericyte coverage of vessels and decreased vessel density (Bohndiek et al., 2015). High tumour sO_2_ was demonstrated to be an early biomarker of radiotherapy response (Rich and Seshadri, 2016) and could predict which tumours would respond to radiotherapy in mouse models of head and neck cancer (Costa et al., 2018). These studies demonstrate the potential of PAI in predicting and monitoring tumour response, which could assist with patient stratification and inform therapeutic strategies.

Exogenous contrast agents can also be used to assess metabolic changes in the hypoxic TME. Amine-oxide probes can give a direct measure of tissue hypoxia (Knox et al., 2017), complementing the assessment of vascular features with endogenous PAI. The pH can be evaluated (Chen et al., 2015) using ratiometric changes in the optical absorption coefficient of a given probe (Chatni et al., 2011; Jo et al., 2017), allowing visualisation of the acidic pH of the TME (Fig. 3C), particularly in the tumour core (Jo et al., 2017). The ability to image several of these features within a single tumour simultaneously, by acquiring data at multiple wavelengths, is one of the unique advantages of PAI that could yield tremendous insight into the dynamic TME interactions.

In a clinical context, application of PAI to monitor vascular features of the TME have focussed on breast cancer, where PAI can be combined with existing ultrasound imaging approaches. Hand-held PAI probes, similar to existing ultrasound probes, have been used in clinical trials to demonstrate higher THb in tumours compared to normal tissue (Diot et al., 2017) and an increase in abnormal features such as vessels radiating from the tumour mass (Neuschler et al., 2018). Similar to standard X-ray mammography, the Twente photoacoustic mammoscope compresses the breast between a glass window and a flat ultrasound transducer matrix (Manohar et al., 2005). This system identified malignant lesions that displayed high PA contrast independent of breast density (Heijblom et al., 2016), but specificity remains a concern (Heijblom et al., 2016). Bespoke hemispherical transducer arrays, which form a cup that surrounds the whole human breast, have also been developed. These systems have become highly sophisticated in recent years, revealing the detailed vessel networks in the breast (Kruger et al., 2016; Lin et al., 2018; Toi et al., 2017; Yamaga et al., 2018). A pivotal study recently demonstrated that, when the patient holds their breath during the scan, thereby decreasing motion artefacts, detailed vascular features can be resolved to a depth of up to 4 cm (Fig. 3D) (Lin et al., 2018). These studies demonstrate how PAI could be utilised to diagnose and stage breast cancers based on vascular TME features.

## Photoacoustic imaging of the ECM and lipid composition

To date, the application of PAI to detect fibrosis and lipids *in vivo* has been focused mainly on diseases beyond cancer, such as Crohn's disease (Lei et al., 2016; Zhu et al., 2018) and atherosclerosis (Cao et al., 2018; Jansen et al., 2014; Wang et al., 2012; Wu et al., 2015); however, early studies show promise for its application in the TME.

In preclinical studies of chronic fibrotic diseases such as Crohn's disease, collagen content has been probed at wavelengths above 1000 nm to detect fibrotic disease in *ex vivo* and *in vivo* rat intestine (Lei et al., 2016; Zhu et al., 2018), with results correlating to histological analyses. In a pivotal PAI clinical trial of 108 patients, active and non-active Crohn's disease was distinguished by measuring THb levels in the intestinal wall, bypassing the need to visualise collagen directly (Knieling et al., 2017). In a preclinical model of liver fibrosis, the PAI signal was higher in fibrotic livers compared to control livers, although the individual contributions of collagen, haemoglobin and possibly other chromophores to the signal could not be determined with the single 808 nm wavelength used in this study (van den Berg et al., 2016). Visualising collagen with PAI is challenging, as light above 1000 nm will be absorbed by lipids, water and collagen in the skin, ultimately leading to a decreased signal from deeper tissues (Zhu et al., 2018), which may explain the lack of clinical studies to date. To enable clinical translation, the application of depth- and wavelength-dependent light fluence (Box 1) correction models could be evaluated, although the application of these models *in vivo* remains challenging due to limited knowledge of the spatial distribution of optical parameters in living tissue (Brochu et al., 2017; Cox et al., 2012). Alternative methods of light delivery directly to the tumour site using fibre optics may also circumvent such challenges with minimally invasive rather than completely non-invasive imaging.

Monitoring the production or activity of MMPs in the TME could provide a diagnostic or predictive biomarker of metastasis and has also been achieved with PAI using exogenous contrast agents (Dragulescu-Andrasi et al., 2013; Levi et al., 2013; Yin et al., 2019). The production of MMPs has been monitored preclinically, with a probe cleaved by MMP-2 and MMP-9 giving an increase in PAI signal upon cleavage in preclinical models of follicular thyroid carcinoma (Levi et al., 2013), which express high levels of MMP-2, MMP-7 and MMP-9 compared to benign thyroid adenomas. Recently, a similar PAI probe design was also shown to quantify MMP-2 expression in mouse mammary tumours (Yin et al., 2019). Given the many roles of MMPs in tumour development and progression (Egeblad and Werb, 2002), monitoring their production and activity *in vivo* is of interest.

PAI of lipids has been mainly applied to monitor atherosclerotic plaques using an intravascular-imaging catheter in either rabbit aorta or *ex vivo* human coronary arteries (Cao et al., 2018; Jansen et al., 2014; Wang et al., 2012; Wu et al., 2015), although these studies lack quantification. Nonetheless, using the catheter bypasses the issue of absorption of light above 1000 nm in the skin, allowing visualisation of lipid distribution in the vessel wall. Single-wavelength imaging visualised relative lipid content in diseased and non-diseased states (Cao et al., 2018; Wang et al., 2012), whereas dual- or multi-wavelength imaging identified cholesterol derivatives in the plaques (Jansen et al., 2014; Wu et al., 2015). This methodology could also be used to monitor lipid content in tumours that can be accessed endoscopically, such as colorectal cancer, which has been shown to be driven by fatty acid synthesis pathways (Zhan et al., 2008).

**Fig. 4.**
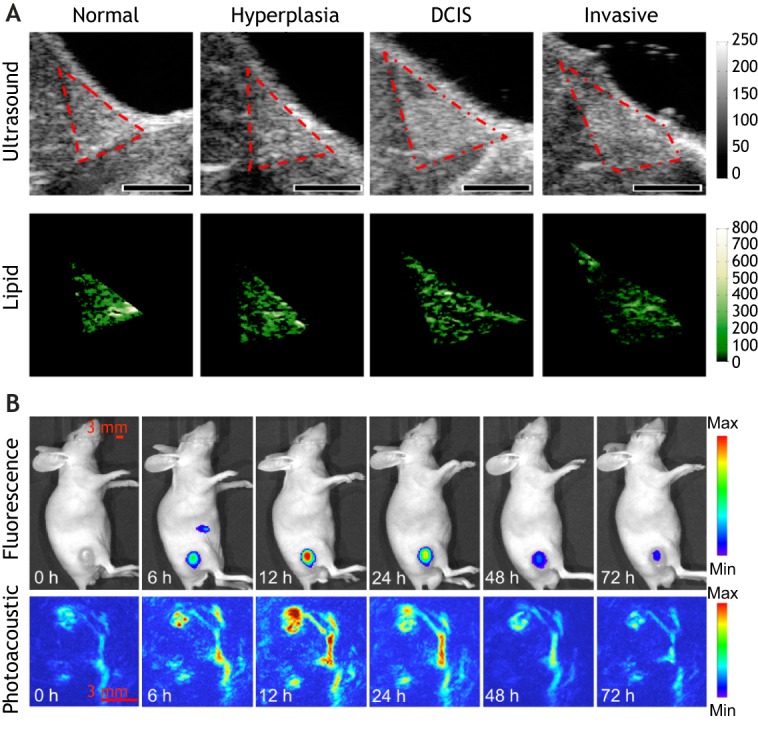
**Example photoacoustic images of lipid content and immune cell tracking.** (A) Representative transverse B-mode ultrasound images (top row) and PAI performed at multiple wavelengths to reveal lipid content (bottom row) of four histologies [normal, hyperplasia, ductal carcinoma *in situ* (DCIS) and invasive carcinoma] from distinct animals of a transgenic mouse model of breast cancer progression. Lesion severity increases from left to right. Regions of interest are outlined in red on B-mode images. Scale bars: 2 mm. Reproduced with minor formatting changes from Wilson et al. (2014). (B) *In vivo* imaging of near-IR-797-labelled T cells in a mouse sarcoma model displaying infiltration of T cells over time with a peak at 12 h and subsequent decline up until 72 h post-adoptive transfer. Copyright Wiley-VCH Verlag GmbH & Co. KGaA. Reproduced from Zheng et al. (2018) with permission, with minor formatting changes. This image is not published under the terms of the CC-BY licence of this article. For permission to reuse, please see Zheng et al. (2018).

As mentioned earlier, lipids play a vital role in the breast TME. In transgenic mice, *in vivo* multi-wavelength PAI has demonstrated a decrease in lipid content with increasing tumour stage (Fig. 4A), presumably as fatty structures in the breast are replaced with fibrotic structures (Wilson et al., 2014). The clinical potential of lipid PAI in breast cancer has been demonstrated using a hand-held multispectral optoacoustic tomography probe. This study showed that lipid structures nearby tumours were disrupted, perhaps as tumours invaded the surrounding healthy adipose tissue (Diot et al., 2017). Additionally, signals attributed to lipids were higher in larger tumours compared to smaller ones, but how this affected the biology of these patients' tumours remains to be elucidated (Diot et al., 2017).

## Photoacoustic imaging of immune cell infiltration

Exogenous contrast agents can theoretically label any cell type of interest and track these cells in the TME. Strong absorbers with little or no fluorescence provide the best contrast because the absorbed energy can be converted efficiently into ultrasound signals rather than being re-emitted as fluorescence. Fluorophores with a low-quantum yield have also proved successful in providing photoacoustic contrast. Fluorescently labelled T cells were first imaged with PAI *ex vivo* (Tzoumas et al., 2014). Since then, exogenous T-cell migration into subcutaneous tumours *in vivo* was monitored with PAI after labelling with a near-infrared fluorescent dye (Fig. 4B) (Zheng et al., 2018). This is one of the first reports of immune cell tracking with PAI and demonstrates the capability of PAI to longitudinally monitor immune cells in the TME at depths beyond the optical diffusion limit (Box 1). Organic nanoparticles and small-molecule dyes have been used as contrast agents to label and track injected stem cells *in vivo* with PAI (Filippi et al., 2018; Yin et al., 2018), a method that could be applied to numerous cell types (Meir et al., 2014; Weber et al., 2016). Such pre-loading of cells with nanoparticles avoids macrophage phagocytosis, which is an issue with systemic delivery of nanoparticles (Weber et al., 2016). In addition to cell labelling, the use of PAI with genetic reporters has expanded (Brunker et al., 2017). For example, cancer cells engineered to produce eumelanin via tyrosinase reporter expression allowed detailed 3D imaging of tumour growth *in vivo* (Jathoul et al., 2015). The extent to which this more invasive genetic approach would be tolerated by the immune system in a clinical setting has yet to be established. Another challenge with reporter genes is the interference of background signal from endogenous chromophores such as haemoglobin, which could be overcome with the use of photoswitchable proteins whose absorption spectra changes upon illumination with a specific wavelength (Yao et al., 2015). Reporter gene technology could be applied to track immune cells in the future (Brunker et al., 2017).

## Conclusions

We have moved into an age of cancer biology research where tumours are considered not as isolated masses of cancer cells but as ‘organs’ with multiple cell types supporting growth and development. The complex contribution of the TME to tumour evolution is now well recognised as influencing cancer cell gene expression, growth, survival, motility, local invasion and metastasis. There are also dynamic interactions between different TME features that regulate processes such as blood vessel outgrowth that we are just beginning to understand.

Given the importance of the TME in tumour development and progression, it is surprising that our ability to image TME features is lacking both in preclinical models and in clinical applications. Many existing imaging modalities have been used to visualise vasculature, hypoxia, fibrosis, lipid synthesis and immune cells (Table 1); however, they tend to carry high cost, require injection of contrast agents, resolve just one feature at a time, and exhibit poor spatial and temporal resolution. PAI is a relatively low-cost modality that has the potential to overcome some of these shortfalls; for example, it can visualise multiple features by acquiring data at multiple wavelengths to separate both endogenous contrast from haemoglobin, collagen and lipids, and visualise injected exogenous contrast agents for features such as acidity, enzyme activity and immune cells, generating a complete picture of the TME. PAI spatial resolution scales with penetration depth, reaching ∼100 μm at ∼3 cm depth, which is advantageous for exploring relationships between the different TME features preclinically.

The studies reviewed here indicate that PAI can inform on TME features in preclinical models and holds promise for clinical translation. Currently, further technical and biological validation of PAI biomarkers is needed to increase uptake of the modality in studies of TME biology or clinical evaluation of TME features (Waterhouse et al., 2019). In terms of technical validation, some studies have reported on standardisation of data acquisition and analysis (Abeyakoon et al., 2018; Bohndiek et al., 2013; Joseph et al., 2017; Martinho Costa et al., 2018; Neuschmelting et al., 2016). Establishing precise and accurate PAI biomarker measurements will provide confidence, while development of standardised stable test objects, or ‘phantoms’, that can be applied in a multi-centre setting is vital for routine quality assurance and control (O'Connor et al., 2017). Additionally, correcting for spectral distortions of illumination light as it passes through tissue would allow absolute quantification of optically absorbing molecules, but remains a significant challenge to apply *in vivo* and hence is an active area of research in the field (Brochu et al., 2017; Cox et al., 2012).

In terms of biological validation, the extensive use of cell-line-derived cancer models in PAI studies limits the clinical applicability of the work due to the stark genomic and phenotypic differences between clonal cell lines and patient samples (Choi et al., 2014). More clinically relevant models, such as patient-derived xenografts, are gaining popularity (Bruna et al., 2016), although the use of immunocompromised mice limits studies of the immune infiltrate in the TME and of the regulation of angiogenesis by immune cells (De Palma et al., 2017). Humanising the immune system of immunocompromised mice is underway in many laboratories (Morton et al., 2016; Shultz et al., 2007), and applying PAI in these more advanced models would help to advance the imaging modality. Further insights can be obtained by correlating *in vivo* PAI data with other well-validated *in vivo* imaging methods as well as *ex vivo* analyses such as immunohistochemistry and biochemical assays. In the few studies where this has been achieved preclinically, PAI provided *in vivo* biomarkers of vascular maturity and function that correlate with hypoxia (Gerling et al., 2014; Quiros-Gonzalez et al., 2018; Tomaszewski et al., 2017). Additionally, validation of photoacoustic mammography in patients showed an *in vivo* distribution of haemoglobin signal that had good colocalisation with DCE-MRI and correspondence with vascular patterns measured *ex vivo* (Heijblom et al., 2015).

Most clinical studies discussed in this Review are observational and/or conducted in a limited number of patients (Diot et al., 2017; Heijblom et al., 2016; Kruger et al., 2016; Lin et al., 2018; Toi et al., 2017; Yamaga et al., 2018). Their purpose was to investigate how PAI biomarkers could be used in disease diagnosis or assessment of disease severity, for example in differentiation between benign and malignant disease. The best-developed application in this regard, including multi-centre studies, is in breast cancer. PAI could be easily combined with ultrasound imaging, which is conventionally used in breast cancer patient management. PAI has been demonstrated to accurately downgrade benign masses (Menezes et al., 2018; Neuschler et al., 2018) with higher specificity than ultrasound imaging (Neuschler et al., 2018), which could reduce unnecessary biopsies and follow-up appointments, reducing patient distress and healthcare costs. As yet, PAI has not been used in clinical decision-making and the results of these studies did not affect the diagnostic pathways of the patients involved; radiologists interpreting the photoacoustic images were blinded to the rest of the diagnostic work-up. Other superficial malignancies could also be monitored with PAI. For example, high-resolution PAI is capable of visualising microvasculature in human skin and could be applied to monitor the pathological neovascularisation of melanoma (Hindelang et al., 2019; Omar et al., 2015). Photoacoustic endoscopy is expanding, bypassing limitations in the penetration depth, so could be used to monitor angiogenesis in gastrointestinal tract cancers or cervical cancer with further technological advances (Li et al., 2018; Qu et al., 2018; Yoon and Cho, 2013). Large-scale clinical trials are also being planned for inflammatory conditions (Knieling et al., 2017).

To conclude, PAI can visualise multiple TME features with a single modality; high spatiotemporal resolution, low cost, use of non-ionising radiation and non-invasive properties with potentially easy integration into existing ultrasound systems make PAI an attractive option for monitoring dynamic TME features not only in a preclinical setting but also throughout a patient's treatment regime, from diagnosis and staging to monitoring treatment response.
